# Alcohol-induced Cushing syndrome: report of eight cases and review of the literature

**DOI:** 10.3389/fendo.2023.1199091

**Published:** 2023-06-20

**Authors:** Asif Surani, Ty B. Carroll, Bradley R. Javorsky, Hershel Raff, James W. Findling

**Affiliations:** ^1^ Departments of Medicine, Medical College of Wisconsin, Milwaukee, WI, United States; ^2^ Department of Medicine. Clement J. Zablocki Veterans Affairs Medical Center, Milwaukee, WI, United States; ^3^ Departments of Surgery, Medical College of Wisconsin, Milwaukee, WI, United States; ^4^ Departments of Physiology, Medical College of Wisconsin, Milwaukee, WI, United States; ^5^ Cardiovascular Center, Medical College of Wisconsin, Milwaukee, WI, United States

**Keywords:** hypercortisolemia, Cushing syndrome, phosphatidylethanol (PEth), alcohol abuse disorder, cortisol

## Abstract

**Introduction:**

Alcohol-induced hypercortisolism (AIH) is underrecognized and may masquerade as neoplastic hypercortisolism [Cushing syndrome (CS)] obscuring its diagnosis.

**Objective and methods:**

In order to characterize AIH, we performed a chart review of eight patients (4 males and 4 females; 2014-2022) referred for evaluation and treatment of neoplastic hypercortisolism — six for inferior petrosal sinus sampling, one due to persistent CS after unilateral adrenalectomy, and one for pituitary surgery for Cushing disease (CD). Five underwent dDAVP stimulation testing.

**Results:**

All eight patients had clinical features of hypercortisolism and plasma ACTH levels within or above the reference interval confirming hypothalamic-pituitary mediation. All had abnormal low-dose dexamethasone suppression test and increased late-night salivary cortisol. Only one had increased urine cortisol excretion. In contrast to CD, the 5 patients tested had blunted or absent ACTH and cortisol responses to desmopressin. Two had adrenal nodules and one had abnormal pituitary imaging. Most patients underreported their alcohol consumption and one denied alcohol use. Elevated blood phosphatidyl ethanol (PEth) was required in one patient to confirm excessive alcohol use. All patients had elevations of liver function tests (LFTs) with AST>ALT.

**Conclusion:**

AIH is an under-appreciated, reversible cause of non-neoplastic hypercortisolism that is indistinguishable from neoplastic CS. Incidental pituitary and adrenal imaging abnormalities as well as under-reporting of alcohol consumption further confound the diagnosis. Measurement of PEth helps to confirm an alcohol use disorder. Elevations of LFTs (AST>ALT) and subnormal ACTH and cortisol responses to dDAVP help to distinguish AIH from neoplastic hypercortisolism.

## Introduction

Alcohol intake acutely increases cortisol secretion and individuals with active alcohol use disorder have increased indices of cortisol secretion compared to controls (1, 2). The mechanism for this increase is thought to be centrally mediated due to increases in hypothalamic corticotropin releasing hormone (CRH) and ACTH (3, 4). In the late 1970s, the presence of both clinical and biochemical evidence of hypercortisolism in patients with alcohol use disorder was recognized, and resolution of cortisol excess was found to occur within 1-2 months after abstention from alcohol (5, 6). Many individuals with an alcohol use disorder may hide or underreport their alcohol consumption making accurate quantification of alcohol consumption difficult (7). Despite the recognition of alcohol-induced hypercortisolism (AIH) [formerly known as a component of the pseudo-Cushing syndrome] for decades, as well as the increasing prevalence of the alcohol use disorder, there is a dearth of reports of AIH in the last 20 years.

Testing hypothalamic-pituitary-adrenal (HPA) axis function has evolved over the past four decades with the development of more sensitive and specific assays and improved diagnostic thresholds (8, 9). Despite improved testing, diagnostic challenges are often encountered when trying to differentiate AIH from neoplastic hypercortisolism. This is in part due to the overlap of clinical and biochemical findings of AIH and neoplastic hypercortisolism (10). In addition, since chronic or intermittent ACTH hypersecretion may lead to the formation of adrenal nodules (11), it is not surprising that AIH has been associated with adrenal nodular disease (12).

The goal of this case series is to describe, highlight, and raise awareness of clinical, laboratory, and imaging findings of AIH. This study will also provide a review of the existing literature.

## Subjects and methods

This retrospective chart review was approved by the Institutional Review Board of the Medical College of Wisconsin. Patients were referred by other endocrinologists to one of us (JWF) for evaluation and treatment of hypercortisolism (Cushing syndrome) between 2014-2022. The complete case reports are presented as a **Supplemental File** (13). Six patients were referred for consideration of inferior petrosal sinus sampling (IPSS) for the differential diagnosis of ACTH-dependent Cushing’s disease (CD), one was referred in anticipation of pituitary surgery for CD, and one patient who previously had a unilateral adrenalectomy with removal of a benign adrenal adenoma without resolution of hypercortisolism.

**Figure 1:**
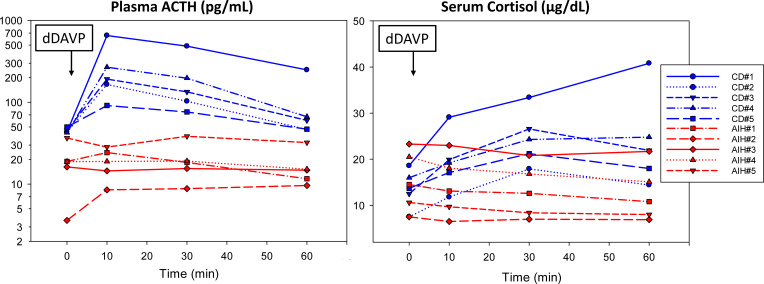
Five reported patients with alcohol-induced hypercortisolism (AIH) lacked a significant ACTH and cortisol response to dDAVP (10 mcg IV) compared to five randomly selected patients with surgically confirmed Cushing disease (CD). For details about each case, please see the **Supplemental File** (13).

Five of the patients with AIH had dDAVP stimulation tests performed. The results were compared to dDAVP stimulation tests in patients with surgically confirmed CD performed between 2019-2022 (**Figure 1**). The dDAVP stimulation test was performed in the morning (before 10:00 am) using an indwelling venous catheter for blood sampling and with the patient supine. Blood samples for measurement of ACTH and cortisol were collected at baseline (0 min), 10, 30, and 60 minutes after the intravenous administration of dDAVP (10 mcg infused over one minute). There were no adverse events, and all patients were instructed to restrict oral fluids to <1.5 liters for 24 hours after the procedure. Although there are no widely accepted criteria for the dDAVP stimulation tests, we and others consider a test to indicate Cushing’s disease if any the following occur: 1) peak ACTH is >70 pg/mL, 2) basal-to-peak ACTH increases >30-35 pg/mL, 3) basal-to-peak ACTH increases >33%, 4) basal-to-peak cortisol increase >20%, or 5) basal-to-peak cortisol increased >4.5 mcg/dL (14–16).

Blood PEth and urine free cortisol were measured by LC-MS/MS at ARUP Laboratories (Salt Lake City, UT). Salivary cortisol was measured by EIA (17). Plasma ACTH assay was measured by immunometric assay (18). Serum cortisol was measured by immunoassay (19).

## Clinical, biochemical, and imaging findings

Four men and four women (24-67 years old) were referred for the confirmation or differential diagnosis of hypercortisolism between 2014-2022 (**Table 1**). Clinical features of these patients are summarized in **Table 1** with detailed case descriptions in **Supplementary Data** (13). Seven of the eight patients had overt physical findings of hypercortisolism including facial rounding with plethora, truncal obesity, increased dorsocervical and supraclavicular fat accumulation, and violaceous striae. One patient with cachexia, myopathy, and edema was thought to have ectopic ACTH due to markedly elevated ACTH (>1000 pg/mL); this patient has been previously reported (18). Five patients were treated for hypertension, 4 patients had diabetes mellitus or prediabetes, and one patient had osteoporosis.

**Table 1:** Clinical and laboratory characteristics of our patients with alcohol-induced hypercortisolism (AIH).

Pt #	Age/Sex	BMI	HTN	DM/ Pre-DM	Alcohol consumption daily (drinks) for duration (years)	LNSC (nmol/L)	Urine free cortisol (µg/day)	Basal ACTH (pg/mL)	DST (cortisol) (µg/dL)	DST (ACTH) (pg/mL)	LFTs (U/L)	CT adrenal
1.	67/M	35.7	Y	Y	3-5 for 20 years	8.2	–	24.2	2.7	<1.5	ALT 34 AST 32	1.2 cm left adrenal nodule
2.	41/F	34.5	Y	Y	6-8 for 4 years	5.6	10.0	8.9	14.6	–	ALT 108 AST 484	–
3.	52/F	46.8	N	N	6-7 for several years (underreported alcohol intake)	9.9	16.1	18.3	6.9	–	ALT 72 AST 149	Unremarkable (hepatomegaly)
4.	50/M	41.6	Y	Y	8-9 for several years ^a^	9.0	15.0	35.1	8.5	1.9	ALT 57 AST 73	Unremarkable (early cirrhosis)
5.	41/M	37.3	Y	Y	7-10 for 4 years	10.2	85.0	23.0	2.0	–	ALT 43 AST 52	Unremarkable (cirrhosis)
6.	33/M	23.0	Y	N	8-12 for 10 years	26.8	11.7	101.0	19.1	9.0	ALT 56 AST 191	3.4 cm right adrenal nodule
7.	24/F	27.7	N	N	Denied	6.6	9.0	28.0	2.2	7.9	ALT 192 AST 288	Unremarkable
8.	59/F	16.2	N	N	8-10 for 25 years	71.6	34.0	14.2^b^	8.9	–	ALT 31 AST 58	–

-, results are not available; LNSC, late-night salivary cortisol; DST, Dexamethasone suppression test; LFTs, Liver function test; AST, Aspartate transferase; ALT, Alanine transaminase.

a PEth levels were elevated (402 ng/ml).

b elevated with Siemens Immulite ACTH assay (>1000 pg/ml) (18).

Reference intervals: LNSC ≤3.2 nmol/L; urine free cortisol ≤45 µg/day; basal ACTH 7.2-63.3 pg/mL; DST cortisol <1.8 µg/dL; AST 10-32 U/L; ALT 8-33 U/L.

None of the patients initially admitted to significant alcohol intake and one patient denied alcohol use entirely. After the diagnosis of AIH was suspected based on other findings (e.g., elevated liver function tests), the patients ultimately acknowledged alcohol use disorder with a daily consumption of anywhere from 4-20 alcoholic drinks. The alcohol consumption of one patient (Case 4) was discovered by an elevated blood PEth. One of the more important clues in the diagnosis of possible AIH was liver function abnormalities in seven of eight patients with the AST consistently higher than the ALT. Three patients had cirrhosis, two of whom were eventually referred for liver transplantation. The patient who had denied any alcohol use was identified after hospitalization for alcoholic hepatitis and symptomatic, acute alcohol withdrawal.

The laboratory findings of disorders of HPA axis function in our patients with AIH were similar to patients with neoplastic hypercortisolism. All patients had elevated late-night salivary cortisol measurements (LNSC) and an abnormal overnight 1 mg dexamethasone suppression test (DST). In contrast, 24-hour urinary cortisol (UFC) was only above the reference interval in one patient. Basal ACTH levels varied (8-101 pg/mL) but none were below the reference interval (7-63 pg/mL); post-DST ACTH levels were <10 pg/mL in the four patients in whom it was measured. Of great importance was that the five patients who were tested had a subnormal plasma ACTH or serum cortisol response to dDAVP stimulation (**Figure 1**).

Imaging studies in some of the patients also created diagnostic uncertainty: one patient had a pituitary lesion and was referred for consideration of pituitary surgery. This was eventually considered to be a Rathke’s cleft cyst with no other identifiable anterior pituitary dysfunction. This patient had normalization of cortisol secretion with alcohol abstention. Two patients had an adrenal nodule, one of whom had a unilateral adrenalectomy without any reduction in hypercortisolism.

Abstinence from alcohol use was recommended in all patients diagnosed with AIH. Long-term follow-up revealed that three patients had normalized their cortisol excess after cessation from alcohol. Two patients were eventually referred for liver transplantation. Detailed biochemical follow-up in the remaining patients is lacking, but to our knowledge, none of the eight patients described here have been diagnosed with neoplastic hypercortisolism.

## Discussion

This report of eight patients with AIH adds significantly to the previous literature. Despite the recent increase in alcohol consumption in the United States (20), there are few recent reports of AIH, and it has been our experience that some clinicians are unaware of AIH. Prior to our case series, we are aware of only 37 patients with AIH reported in the literature between 1976-2022 (**Table 2**). Most of the patients had clinical features of hypercortisolism including facial plethora, moon facies, obesity, increased dorsocervical fat, muscle wasting, and weakness. The actual amount of alcohol consumed was not routinely assessed or estimated. Hypertension was reported in 14 of 17 patients (6, 10, 12, 22, 25–27, 29, 31) and diabetes mellitus/prediabetes in 9 of 11 patients (6, 12, 22, 29, 31) in whom clinical data are available. Liver function data have not been consistently reported; however, 20 of 23 had abnormal liver function tests (5, 6, 10, 22, 23, 26, 27, 29–31) and two patients had biopsy-proven alcoholic cirrhosis (27, 29). Most patients did not have high resolution pituitary or adrenal imaging; however, 2 of 4 patients who had CT of the abdomen had unilateral adrenal nodules (5, 12). Only one patient had a pituitary MRI which was unremarkable (25).

**Table 2:** Clinical and laboratory characteristics of patients with alcohol-induced hypercortisolism (AIH) - Literature review.

Author	Age/Sex	BMI	HTN	DM/ Pre-DM	Basal Serum Cortisol-AM (µg/dL)	Basal ACTH (pg/mL)	Urine free cortisol (µg/day)	DST (cortisol) (µg/dL)	DST (ACTH) (pg/mL)	LFTs (U/L)	Brain Imaging	CT adrenal
Kirkman S (1988) (21)	51/M	21.2	N	N	21.1	81.0	127	20.1	101.0	“Normal”	–	–
Smalls AG (1976) (6)	39/F	–	Y	Y	59.0	–	–	14.0	–	“Double the normal”	–	–
	54/M	–	Y	Y	115.0	–	–	34.0	–	“Double the normal”	–	–
	30/M	–	Y	Y	37.0	–	–	14.0	–	AST 60	–	–
Smals AG (1977) (22)	39/M	–	Y	–	38.0	–	–	3.0	–	AST 75	–	–
	59/M	–	Y	Y	26.0	–	–	2.0	–	AST 24*	–	–
	36/F	–	–	Y	26.0	–	–	3.0	–	AST 27*	–	–
Lamberts SWJ (1979- JAMA) (10)	35/M	23.0	Y	–	–	58.0	–	17.0	–	ALT 33 AST 34 GGT 210	–	–
	49/F	24.6	Y	–	22.0	50.0	–	23.2	–	ALT 13 AST 21 GGT 42*	–	–
Jenkins RM (1981) (23)	59/F	–	N	–	38.0	131.0	265	28.0	–	“Raised AST, GGT, ALP”	Unremarkable	–
Proto G (1985) (24)	Multiple ^a^	–	–	–	Elevated	Normal		“Sufficient suppression”		“Normal-slightly elevated”	Unremarkable	Unremarkable
Kurajoh M (2018) (25)	63/M	21.7	Y	–	15.3	56.6	147	9.2	41.3	ALT 8 AST 13	Unremarkable	–
Endo Y (1986) (26)	26/M	–	Y	–	16.1	64.0	–	–	–	ALT 89 AST 42 GGT 101	–	Unremarkable
Aron DC (1981) (27)	34/F	–	Y	N	14.8	47.0	62	<4.0	–	AST 80	–	–
Kapcala LP (1987) (12)	62/F	–	Y	Y	14.4	63.0	106	15.6	13.0	“Normal”	Unremarkable	Left adrenal gland mass 1.7x3.0 cm
Coiro V (2000) (28)	28	24.0	–	–	29.9	–	174	6.9	–	–	–	–
	42	28.0	–	–	27.4	–	169	6.2	–	–	–	–
	30	25.0	–	–	39.5	–	237	7.4	–	–	–	–
	37	23.0	–	–	32.5	–	200	5.1	–	–	–	–
	29	24.0	–	–	26.7	–	194	2.9	–	–	–	–
	42	27.0	–	–	28.4	–	188	0.4	–	–	–	–
	40	22.0	–	–	44.2	–	234	6.2	–	–	–	–
	38	22.0	–	–	37.8	–	229	7.4	–	–	–	–
Rees LH (1977) (5)	30/M	–	–	–	45.6	–	–	3.0	–	“Normal-slightly elevated”	–	–
	52/F	–	N	–	61.9	<20.0	–	–	–	ALT 50	–	–
	38/M	–	–	–	426.0	49.0	–	15.6	–	GGT 310	–	“Left adrenal tumor suspected”
	56/F	–	–	–	27.9	–	–	–	–	GGT 59*	–	–
Frajria R (1977) (29)	52/M	–	Y	Y	4.0	–	–	“Elevated”	–	“Moderately disturbed”	–	–
	33/M	–	Y	Y	15.9	–	–	2.8	–	“Moderately disturbed”	–	–
Lamberts SWJ (1979- J Endocrinol.) (30)	24/M	–	–	–	10.3	7.5	–	2.1	–	“Elevated AST/ALT”	–	–
	49/F	–	–	–	22.1	58.0	–	22.7	–	“Elevated ALP/GGT”	–	–
Jordan RM (1979) (31)	35/F	–	Y	Y	–	–	122	5.0	–	“Moderately elevated”	–	–

-, results are not available; DST, Dexamethasone suppression test; LFTs, Liver function test; AST, Aspartate transferase; ALT, Alanine transaminase; ALP, Alkaline phosphatase; GGT, Gamma-glutamyl transferase.

a Total six patients were reported including 2 females (age 19,30) and 4 males (mean age: 36).

Reference interval (RI) for LFTs: AST 10-32 U/L; ALT 8-33 U/L; GGT 0-60 IU/L.

*Abnormal results (authors have cited a different RI compared to the standard RI mentioned above).

DST was abnormal (8 AM serum cortisol >1.8 mcg/dL) in 25 of 26 AIH patients for whom numerical results were reported (5, 6, 10, 12, 21–23, 25, 28–31); UFC excretion was elevated in all 14 AIH patients tested (12, 21, 23, 25, 27, 28, 31). There are very few reports of LNSC in patients with AIH (32). In one series of eight actively drinking patients with hypercortisolism, there was a lack of an increase in plasma ACTH or serum cortisol in response to dDAVP (28). Not surprisingly, the clinical and biochemical features of hypercortisolism normalized after alcohol cessation in all 26 patients for whom there are follow-up data (5, 6, 10, 12, 21–26, 29–31).

With regard to our patients, seven of our eight patients had the diagnosis of hypercortisolism established by a referring endocrinologist and were referred for differential diagnosis of Cushing’s syndrome. Six patients were referred for consideration of IPSS to confirm the presence of an ACTH secreting pituitary tumor. One with an adrenal nodule had undergone unilateral adrenalectomy without resolution of hypercortisolism. Importantly, many patients either underreported their daily alcohol consumption or even denied any alcohol use. The alcohol use disorder was finally revealed in one patient after the discovery of a marked elevation of blood PEth, a known marker of alcohol intake (33).

Many patients with alcohol use disorder have increases in cortisol secretion (2). Hair cortisol (a reflection of cortisol secretion over approximately 3 months) is elevated in patients with alcohol use disorder and normalize after abstinence from alcohol consumption (34). Others have shown increases in different aspects of HPA axis function in individuals with chronic, active alcohol abuse including the cortisol awakening response (35), overnight urine free cortisol (36), and single assessments of serum cortisol (37) and LNSC concentrations (38, 39). Increases in cortisol secretion occur during alcohol withdrawal with normalization of cortisol secretion within one week of abstinence (40).

Alcohol-induced cortisol secretion is centrally mediated by hypothalamic CRH in rats (3, 4). CRH mRNA expression increases in the hypothalamic paraventricular nucleus of rats after alcohol administration (4) and CRH receptor antagonists abolish this response. Furthermore, alcohol intake does not increase plasma corticosterone in hypophysectomized rats (41, 42). Alcohol-induced increases in hypothalamic vasopressin secretion may also contribute to the increase in HPA axis activity since hypothalamic parvocellular and magnocellular derived vasopressin augments the ACTH response to CRH (43). However, Cobb et al. has suggested a non-ACTH mediated increase in adrenal function with alcohol administration (44). Although Elias et al. showed that a large single bolus of alcohol (45) does not increase ACTH or cortisol in normal subjects, others have reported increased late afternoon ACTH and cortisol levels in individual with chronic, active alcohol abuse (2). Altered peripheral metabolism of cortisol in the liver may also contribute to AIH. Lamberts et al. reported a patient with alcohol use disorder with clinical Cushing syndrome and subnormal ACTH levels demonstrating a prolonged half-life of cortisol (30). Another potential mechanism is the induction of 11-beta-hydroxysteroid dehydrogenase Type 1 gene expression and activity in patients with alcohol liver disease (46). Investigators have shown a significant increase in hepatic cortisol production in patients with alcohol-liver disease compared to normal subjects which may contribute to AIH. Nonetheless, if glucocorticoid negative feedback remained intact, normal cortisol levels might be expected unless stimulatory input to the hypothalamus persisted (46).

The laboratory findings in our patients support a centrally mediated mechanism for hypercortisolism in patients with alcohol use disorder. We found non-suppressed ACTH despite hypercortisolism in our patients establishing an ACTH-driven process. The five patients who underwent dDAVP stimulation testing lacked a significant ACTH and cortisol response, distinguishing them from patients with CD (**Figure 1**). Increases in ACTH and cortisol are well described during the dDAVP stimulation in patients with CD (47). This important finding is consistent with the study of Coiro et al., who also reported the absence of an ACTH and cortisol response to dDAVP in AIH (28).

In contrast, the dexamethasone-CRH test is abnormal in individuals with active, chronic alcohol abuse and indistinguishable from patients with neoplastic hypercortisolism (48). It is also important to point out that the dexamethasone-CRH test is abnormal in other forms of non-neoplastic hypercortisolism such as schizophrenia, mania, and certain forms of depression (49–53). The biological explanation for the heterogeneity in the ACTH response to dexamethasone-CRH in the different forms of non-neoplastic hypercortisolism is not known. As CRH is no longer available, it is not currently feasible to perform this test.

As previously reported, one of our patients was initially thought to have ectopic ACTH due to markedly elevated ACTH (>1000 pg/mL) due to an unreliable ACTH assay; she subsequently had a non-elevated ACTH level by a reliable ACTH assay without a significant increase in ACTH or serum cortisol after ovine CRH administration (18).

Our patients exhibited elevated LNSCs, and like other reports of AIH, all had abnormal DSTs. Only one was found to have an elevation of UFC. UFC lacks sufficient sensitivity to detect milder degrees of hypercortisolism (54). Accordingly, we do not routinely suggest that UFC be utilized as an initial screening test for cortisol excess, especially milder cases of cortisol excess (54).

Our patients had varied physical signs of cortisol excess including truncal obesity, plethoric moon facies, wide abdominal violaceous striae, and myopathy. Many of them had other metabolic consequences of hypercortisolism including hyperglycemia, hypertension, and osteoporosis. These findings in concert with abnormal HPA dynamics led to referral to establish the differential diagnosis of ACTH-dependent hypercortisolism - six were referred for inferior petrosal sinus ACTH sampling and one for pituitary surgery because of a pituitary imaging abnormality.

Chronic use of alcohol is a risk factor for the development of osteoporosis, hypertension, and type 2 diabetes mellitus (55–57). It is possible that activation of the HPA axis in patients with a chronic alcohol use disorder may be a contributing factor to these metabolic issues. With the use of more accurate approaches to assess HPA axis activity (e.g., LNSC), it is important to investigate the possible role of intermittent AIH in the genesis of these metabolic problems.

Elevation of liver function tests in our patients with the AST consistently greater than the ALT was an important laboratory hallmark that helped to establish AIH (58, 59). Our patients initially underreported their alcohol intake, and one denied any alcohol consumption. Blood PEth levels as a means of quantifying chronic alcohol consumption helped confirm AIH in the one patient in whom it was measured and would have likely been helpful in all patients had this test been available at the time. PEth is a group of phospholipids formed in the presence of ethanol and is a useful biomarker of alcohol intake. PEth is incorporated into the phospholipid membrane of red blood cells and has a half-life of 4-10 days with a window of detection of 2-4 weeks; the window of detection may be prolonged in individuals who chronically or excessively consume alcohol (60).

Imaging findings in our patients also caused diagnostic confusion. Two patients had adrenal nodular disease, one of whom had persistent hypercortisolism despite a unilateral adrenalectomy. Since ACTH hypersecretion may cause adrenal nodules (11), it is not surprising that they have been reported in AIH (12). Since the presence of adrenal nodules on imaging often triggers the consideration of hypercortisolism, clinicians should be aware of the possibility of AIH particularly in patients without suppression of ACTH below the reference interval, and with abnormal liver function tests.

The limitations of this report include its retrospective nature and the lack of long-term patient follow-up. Of course, abstinence from alcohol use was recommended in all patients. Three patients demonstrated normalization of cortisol excess after cessation from alcohol. To our knowledge, none of our eight patients have been diagnosed with neoplastic hypercortisolism.

In summary, this report highlights the importance of the recognition of AIH when evaluating patients with suspected neoplastic hypercortisolism. AIH may be indistinguishable from neoplastic hypercortisolism: LNSC and DST are abnormal in AIH patients, and some may have adrenal and pituitary imaging abnormalities that confound the diagnostic evaluation. Since many patients may underreport alcohol consumption, it is important to be aware of AIH in patients with ACTH dependent hypercortisolism and particularly in those with elevated liver function tests (AST>ALT). Blood PEth is a diagnostic tool that can help to confirm the presence of excessive alcohol consumption. The dDAVP stimulation may help to distinguish AIH from patients with neoplastic ACTH-dependent hypercortisolism.

## Data availability statement

The original contributions presented in the study are included in the article/**Supplementary Material**. Further inquiries can be directed to the corresponding author.

## Ethics statement

Written informed consent was not obtained from the individual(s) for the publication of any potentially identifiable images or data included in this article.

## Author contributions

All authors listed have made a substantial, direct, and intellectual contribution to the work and approved it for publication.
