# Supplementary material for: Alcohol-induced Cushing syndrome: report of eight cases and review of the literature
Source: Front Endocrinol (Lausanne). 2023 Jun 20;14:1199091. doi: 10.3389/fendo.2023.1199091 (PMC10319132; doi:10.3389/fendo.2023.1199091)
Supplement: Supplementary file 1 [file DataSheet_1.pdf]

CASE 1

67-year-old male with a history of hypertension, low testosterone, and obstructive sleep apnea was found to have ACTH dependent hypercortisolism and was referred for inferior petrosal sinus sampling (IPSS). He had an unexplained weight gain of 20 pounds in the past 8-12 months. He acknowledged that he drinks 3-5 alcoholic drinks daily at the time of his initial consultation. His physical exam showed slight facial rounding, and a generous amount of supraclavicular fat was noted. His late-night salivary cortisol (LNSC) was elevated, and he had an abnormal dexamethasone suppression test (DST). A desmopressin (dDAVP) stimulation test showed no increase in ACTH or cortisol levels (Figure 1). His adrenal imaging showed a 1.2 cm left adrenal nodule. However, repeat imaging showed only diffuse thickening of the left adrenal gland without discrete nodules. His pituitary MRI was unremarkable. He was managed as alcohol-induced hypercortisolism (AIH), and alcohol abstinence was recommended.

CASE 2

41-year-old female was referred for evaluation of ACTH dependent Cushing syndrome and consideration for IPSS. She had a history of chronic pain (on opiates) and hypothyroidism. She presented with an unexplained 75-pound weight gain in the last five years. She complained of fatigue, peripheral lower extremity edema, easy bruising, and hair loss. Her physical examination revealed facial plethora, dorsocervical fat pad, truncal obesity, abdominal striae, and supraclavicular fullness. She had elevated LNSC, abnormal DST, and normal ACTH levels. Brain MRI and CT chest were also unremarkable. In addition, she underwent dDAVP stimulation testing which showed no increase in ACTH or cortisol levels (Figure 1). She initially denied alcohol use; however, upon confrontation, she acknowledged having 6-8 alcoholic drinks daily for the last 4 years. Consultation regarding treatment for her alcohol use disorder was recommended.

CASE 3

52-year-old female was referred for ACTH dependent Cushing syndrome due to a history of depression, obesity, and left hip fracture with low bone mineral density.  She had peripheral neuropathy with frequent falls, and weight gain of 60 pounds in the past 9 months. She also reported hair loss, perioral hair growth, new acne development, and tremors. Examination showed classic Cushingoid features with positive supraclavicular fat pad, facial rounding, skin thinning, and violaceous abdominal striae. Biochemical evidence of hypercortisolism was confirmed with elevated LNSC and abnormal DST. Basal ACTH was within normal limits. MRI pituitary was normal and IPSS was considered; however, a dDAVP stimulation test yielded no increase in ACTH or cortisol (Figure 1). Her abdomen CT only revealed hepatomegaly. She had initially reported alcohol intake of 3-4 drinks daily for greater than 10 years. However, after an admission for liver failure and anasarca, her husband reported more than 6 alcoholic drinks daily.

CASE 4

50-year-old male was referred for IPSS for the differential diagnosis of ACTH dependent hypercortisolism. He presented with increasing fatigue, difficulty concentrating, muscle weakness, and unspecified weight gain. His physical exam revealed wide violaceous striae, pedal edema, increased supraclavicular fullness, and truncal obesity. He had an elevated LNSC along with an abnormal DST. Diagnostic imaging revealed a normal pituitary gland, and CT abdomen without contrast did not reveal

any adrenal mass. A dDAVP stimulation test yielded no ACTH or cortisol response (Figure 1). He initially reported only 1-2 alcoholic drinks daily. However, a blood phosphatidylethanol (PEth) level was elevated to 402 ng/mL (>200 ng/ml is consistent with heavy alcohol consumption). After further questioning, he admitted to chronic alcohol abuse with 8-9 alcoholic drinks daily for greater than 10 years. Appropriate counseling and treatment for his alcohol use disorder were recommended.

CASE 5

41-year-old male was referred for pituitary surgery for probable Cushing disease. He had a 5-year history of obesity with an 80 pound weight gain, type 2 diabetes mellitus, depression, gynecomastia, decreased libido, easy bruising, muscle weakness, and easy fatigability. His physical exam was significant for facial rounding, increased supraclavicular fat pad, bilateral gynecomastia, and wide violaceous striae. His LNSC was elevated, and he had an abnormal DST. CT scan of the abdomen showed normal adrenal glands, but cirrhosis was identified. His pituitary MRI was significant for anterior pituitary cyst eccentric to the right measuring 8.1 x 5.7 x 4.4 mm most consistent with a Rathke's cleft cyst. There was no increase in ACTH or cortisol after dDAVP stimulation (Figure 1). He readily acknowledged consuming 7-10 alcoholic drinks daily for the last 4 years. Accordingly, the diagnosis of AIH was confirmed. Four months after his alcohol cessation, his LNSC had normalized.

CASE 6

33-year-old male was referred for evaluation of persistent Cushing syndrome after removal of a 3.4 cm right adrenal adenoma. He presented with weakness, easy bruising, rapid weight gain, facial rounding, and striae. After unilateral adrenalectomy, his hypercortisolism persisted with elevations of LNSC and abnormal DST. A course of mifepristone therapy provided no significant improvement in clinical symptoms and biochemical markers. Furthermore, MRI of the pituitary and CT scan of chest/abdomen/pelvis did not show any evidence of an ACTH secreting neoplasm. An ovine CRH stimulation test demonstrated no increase in ACTH or cortisol. The diagnosis of AIH was considered due to liver function abnormalities (AST>ALT), cerebral atrophy, peripheral neuropathy, and ACTH elevation on repeat testing. After an intervention, he acknowledged binge drinking and an alcohol use disorder (8-12 drinks daily); he had denied alcohol intake during prior visits. Subsequently, he had several admissions due to alcohol related complications and was referred for liver transplantation due to hepatic failure from alcohol induced cirrhosis.

CASE 7

24-year-old female with a history of elevated transaminases and depression presented due to unexplained 30-pound weight gain in the past 1 year. Physical examination revealed classic Cushingoid features including facial plethora, dorsocervical fat, and ecchymosis. Her labs were consistent with hypercortisolism with elevated LNSC and abnormal DST. Imaging of pituitary and adrenal glands were unremarkable. She was referred to our center for consideration of IPSS. During her workup, due to an elevated AST>ALT, the diagnosis of AIH was suspected. Although she denied any alcohol use, six months after her evaluation, she was admitted to another academic institution with alcohol intoxication and alcoholic hepatitis. Abstinence of alcohol resulted in resolution of her clinical features of hypercortisolism.

CASE 8

59-year-old female was referred for consideration of ectopic ACTH secretion due to a plasma ACTH level >1000 pg/mL. She had a history of alcohol use disorder (8-10 drinks daily for the last 25 years), stage 4 cirrhosis with portal hypertension, and chronic hyponatremia. She complained of easy bruising, severe muscle atrophy, weakness, and substantial lower extremity edema. On physical examination, she was cachectic with excessive lanugo hair growth, multiple ecchymoses on her arms, severe proximal muscle weakness, and 2+ pretibial edema. LNSC was elevated, and she had an abnormal DST. Although the basal ACTH level was >1000 pg/mL (Siemens Immulite ACTH), measurement of ACTH using a more reliable assay (Roche platform Cobas ACTH) demonstrated normal basal ACTH concentration without a significant increase after ovine CRH stimulation. Her pituitary MRI was normal and chest CT did not reveal any lesions. After 6 months of abstinence from alcohol, she was approved for liver transplantation. Repeat laboratory studies showed normalization of LNSC and marked clinical improvement of her signs and symptoms of hypercortisolism. (Case previously reported and included here for completeness – see Greene LW, Geer EB, Page-Wilson G, Findling JW, Raff H. Assay-Specific Spurious ACTH Results Lead to Misdiagnosis, Unnecessary Testing, and Surgical Misadventure-A Case Series. J Endocr Soc. 2019 Feb 20;3(4):763-772. doi: 10.1210/js.2019-00027. PMID: 30963134; PMCID: PMC6446888.)
